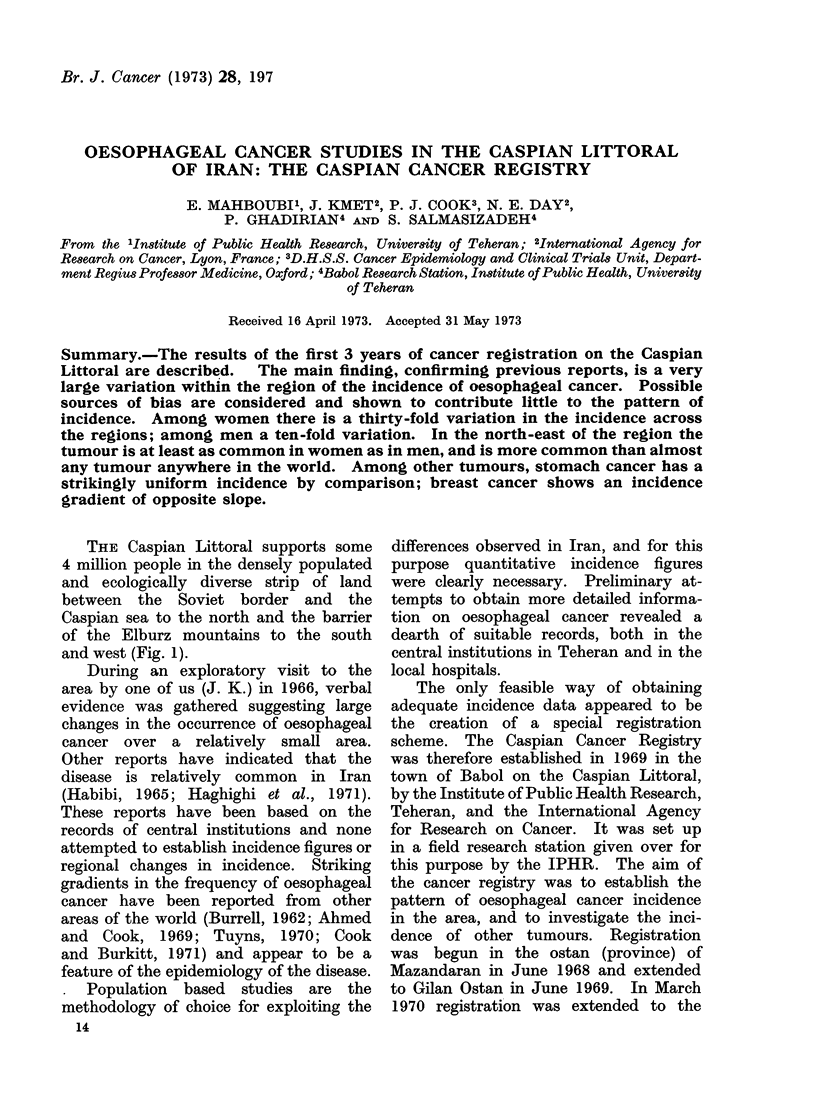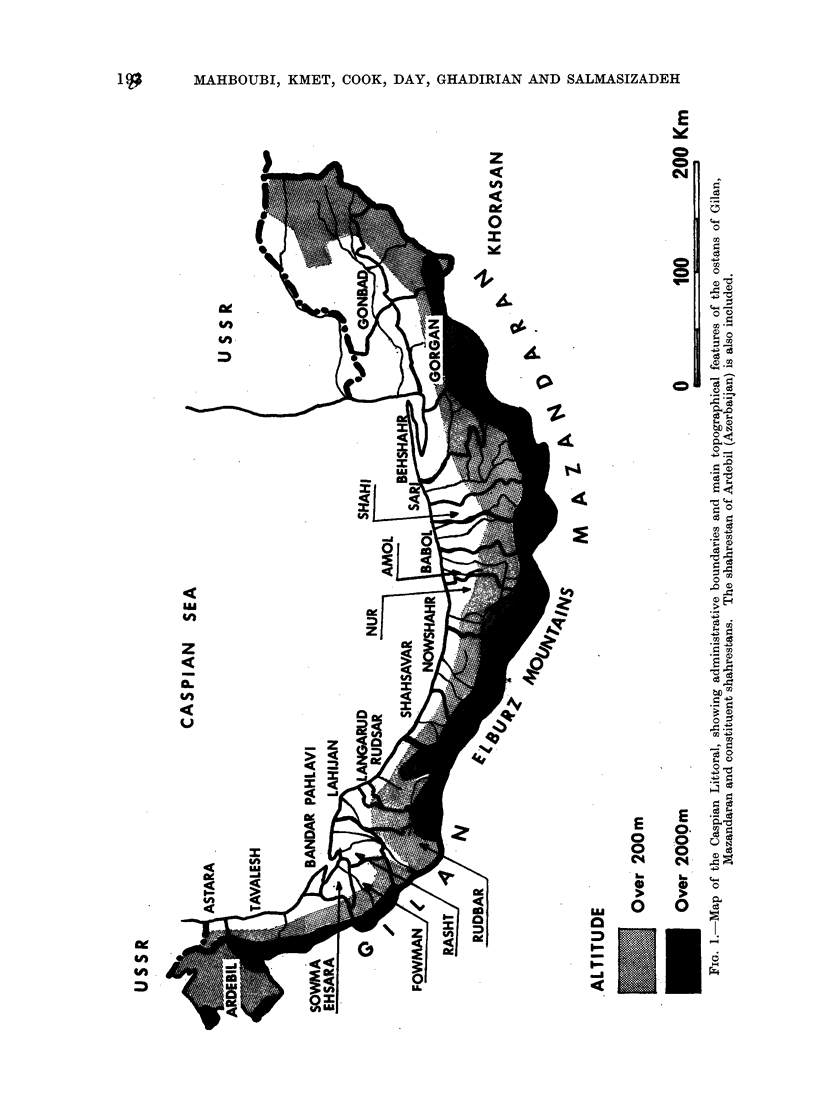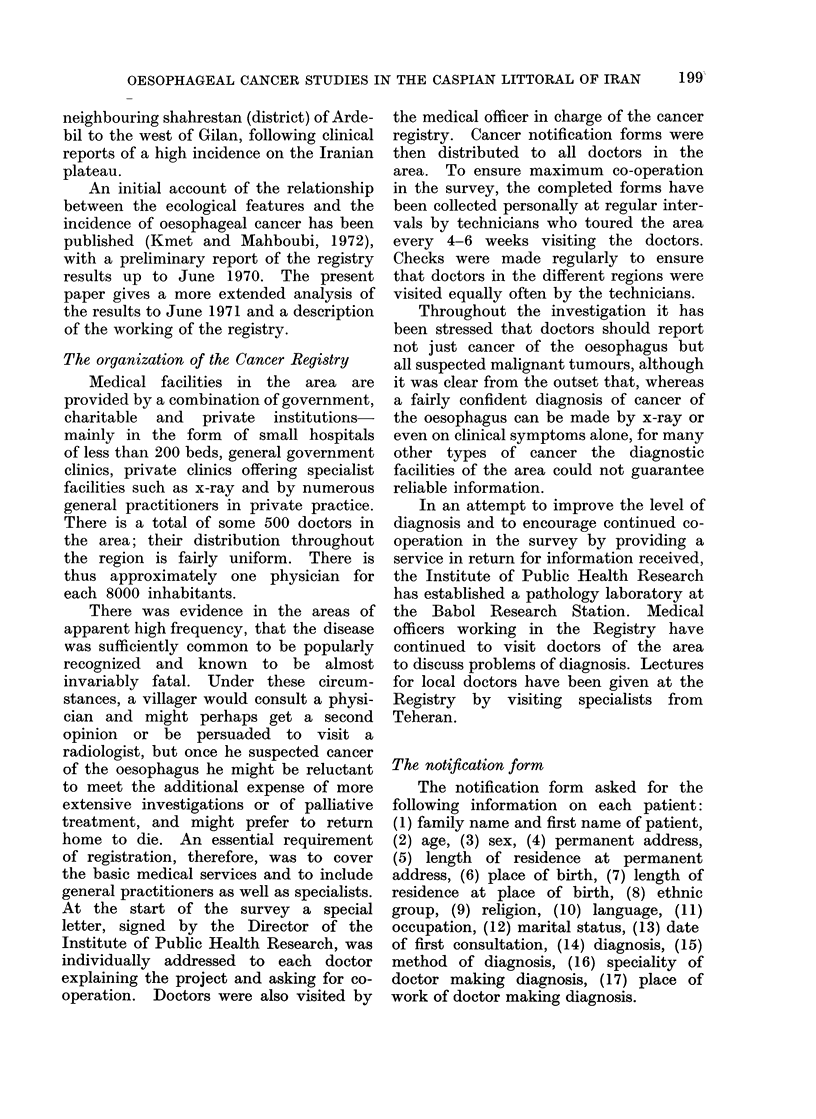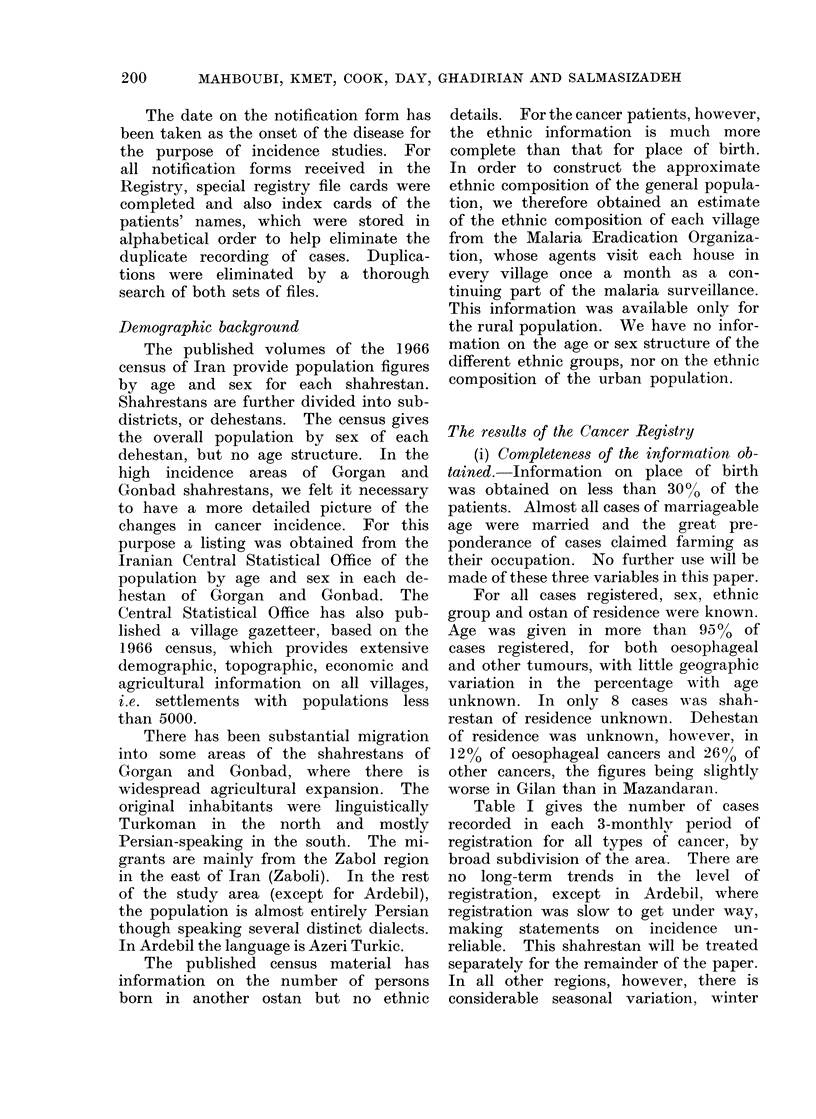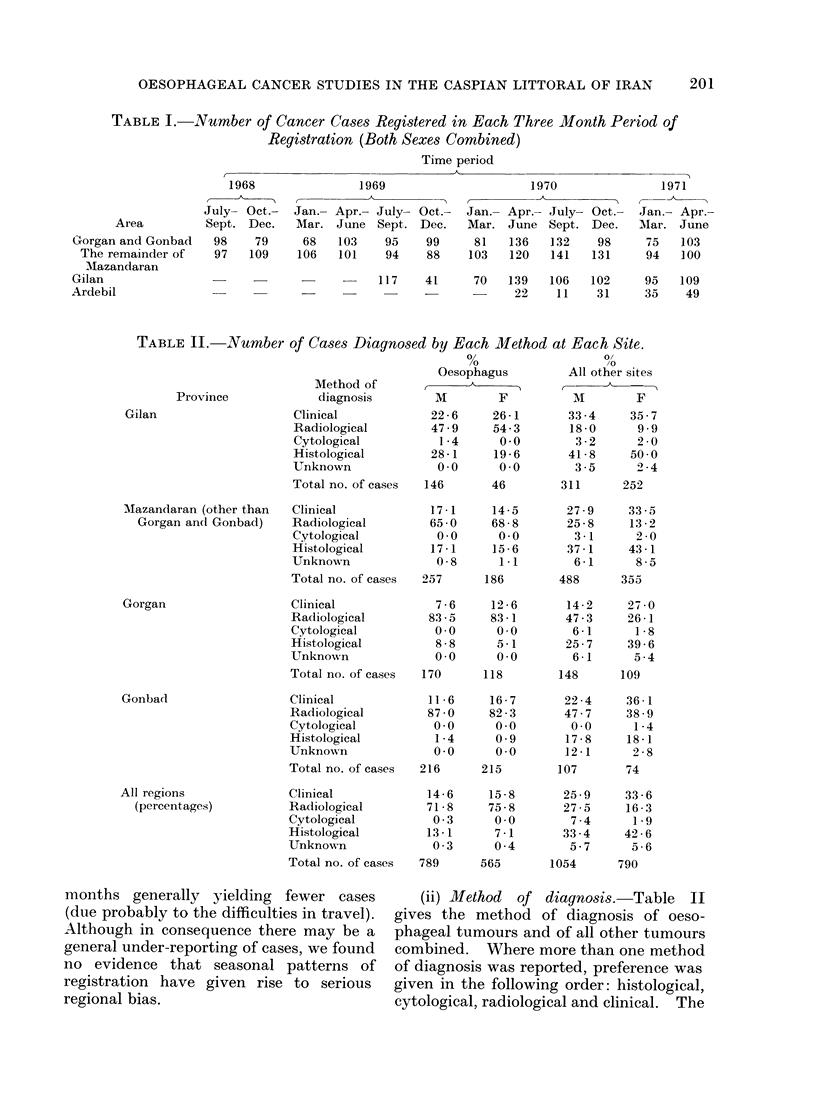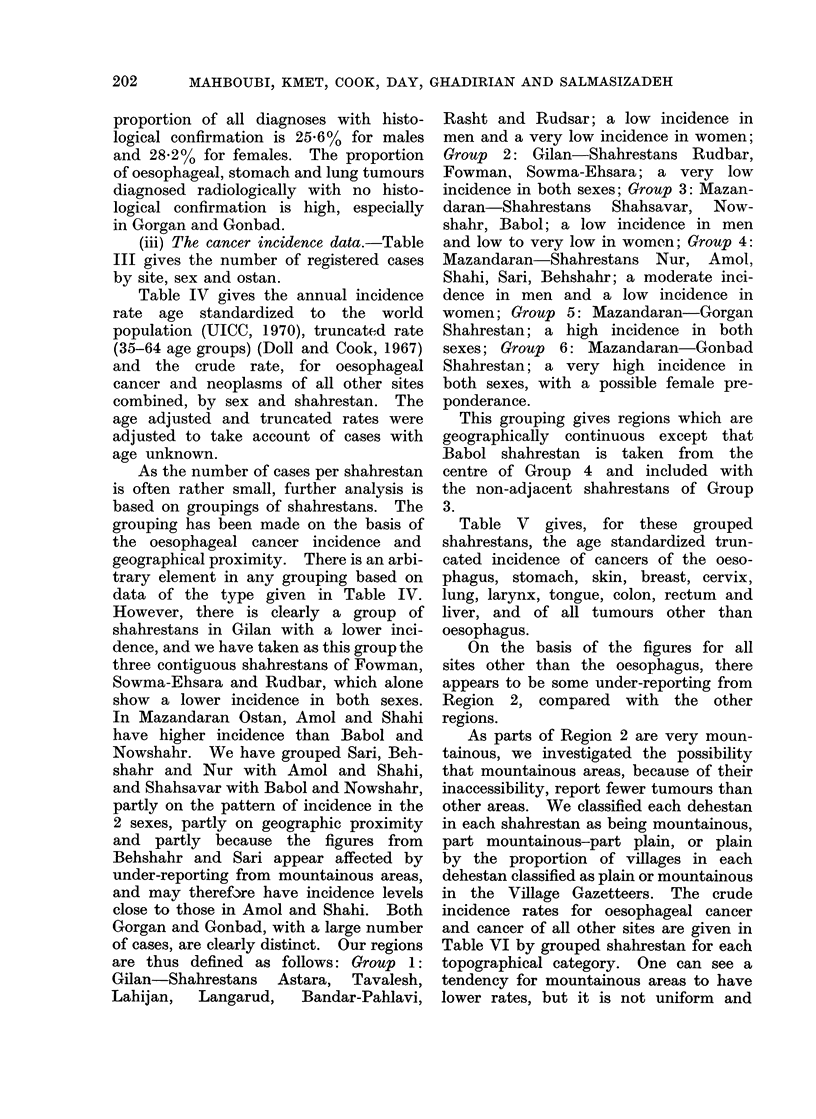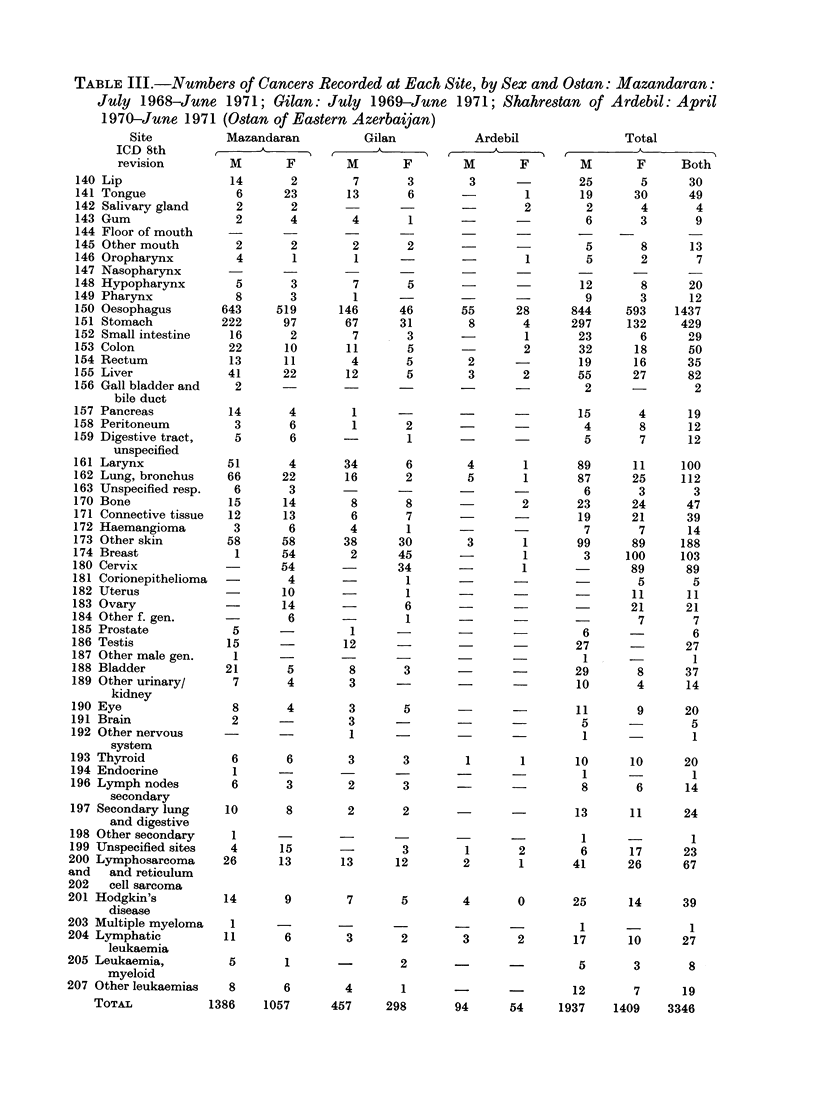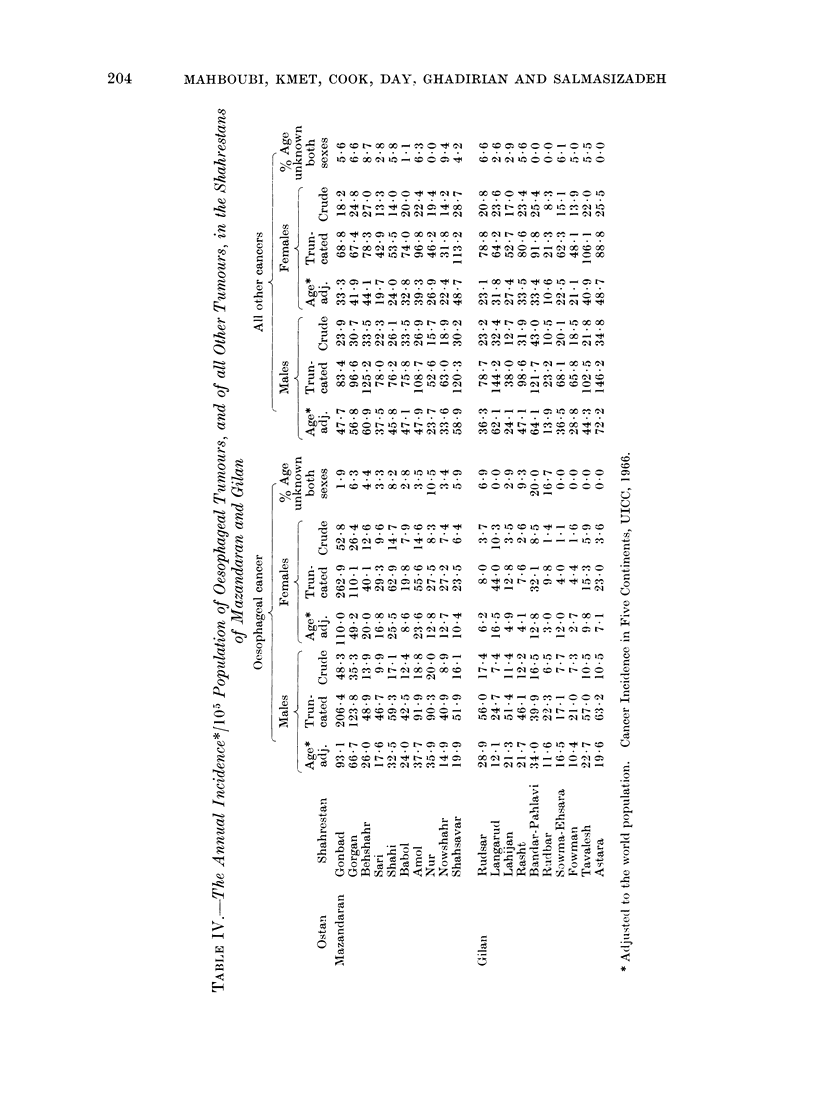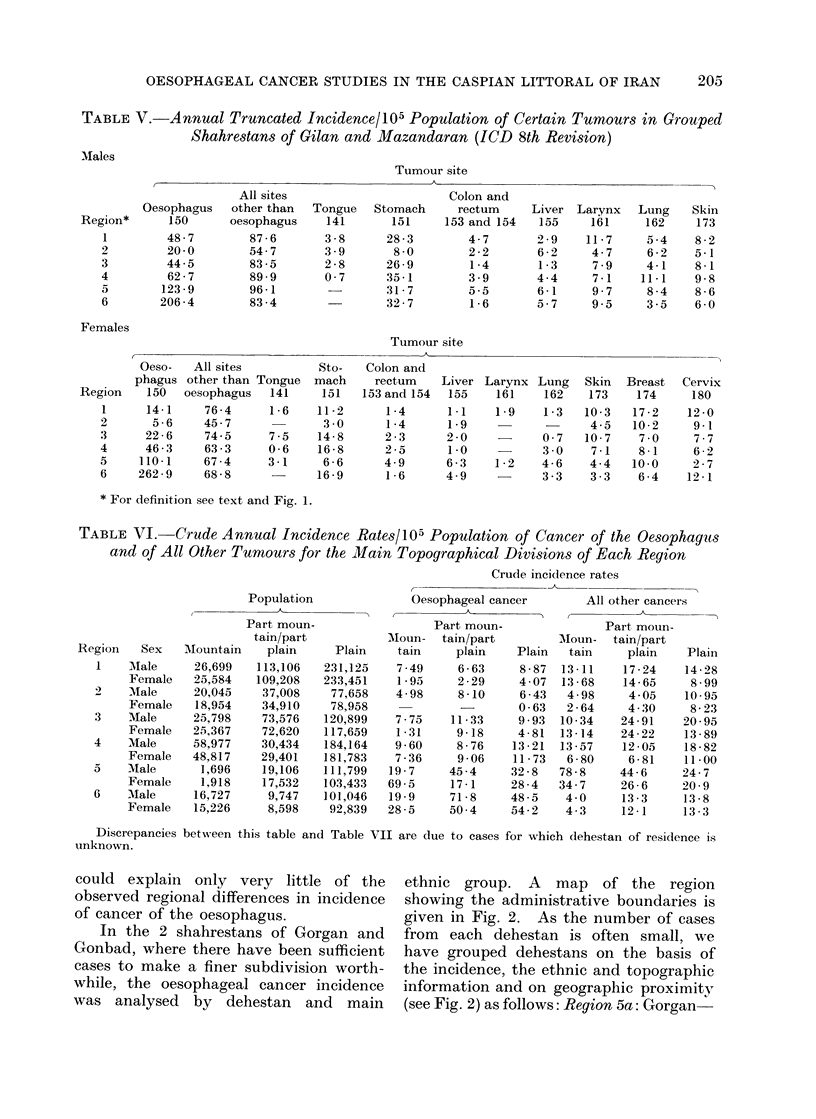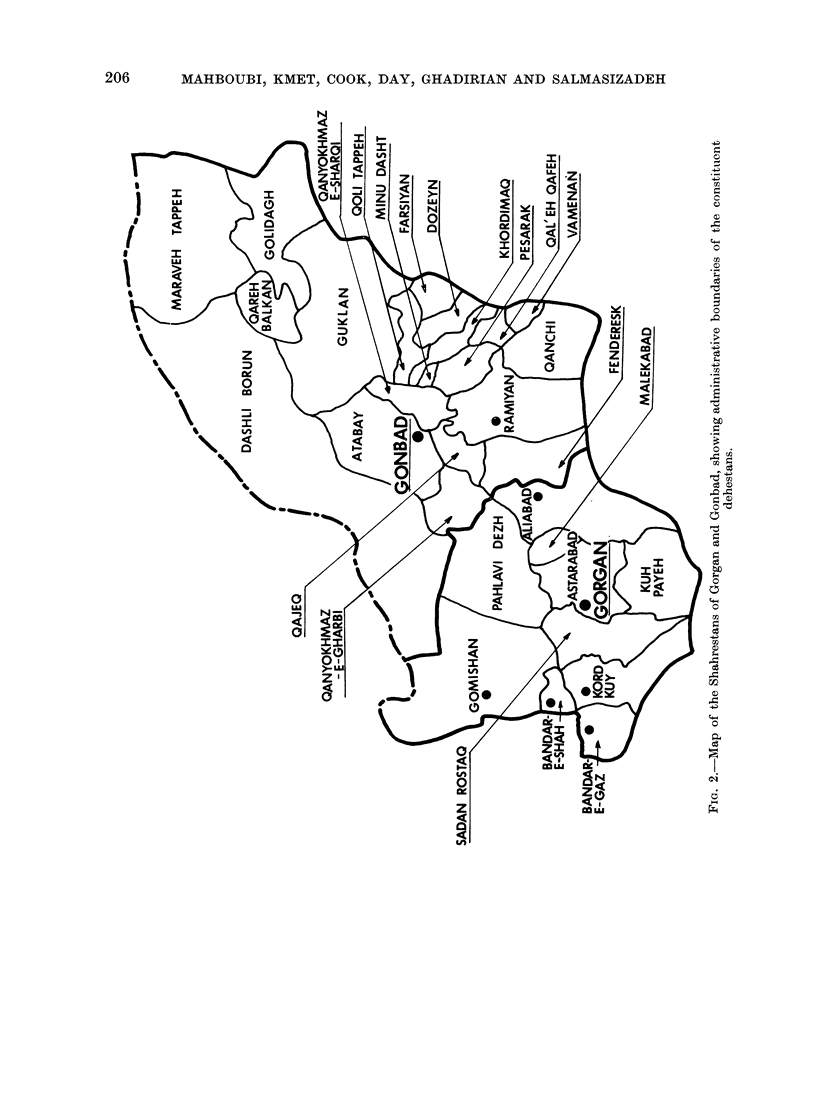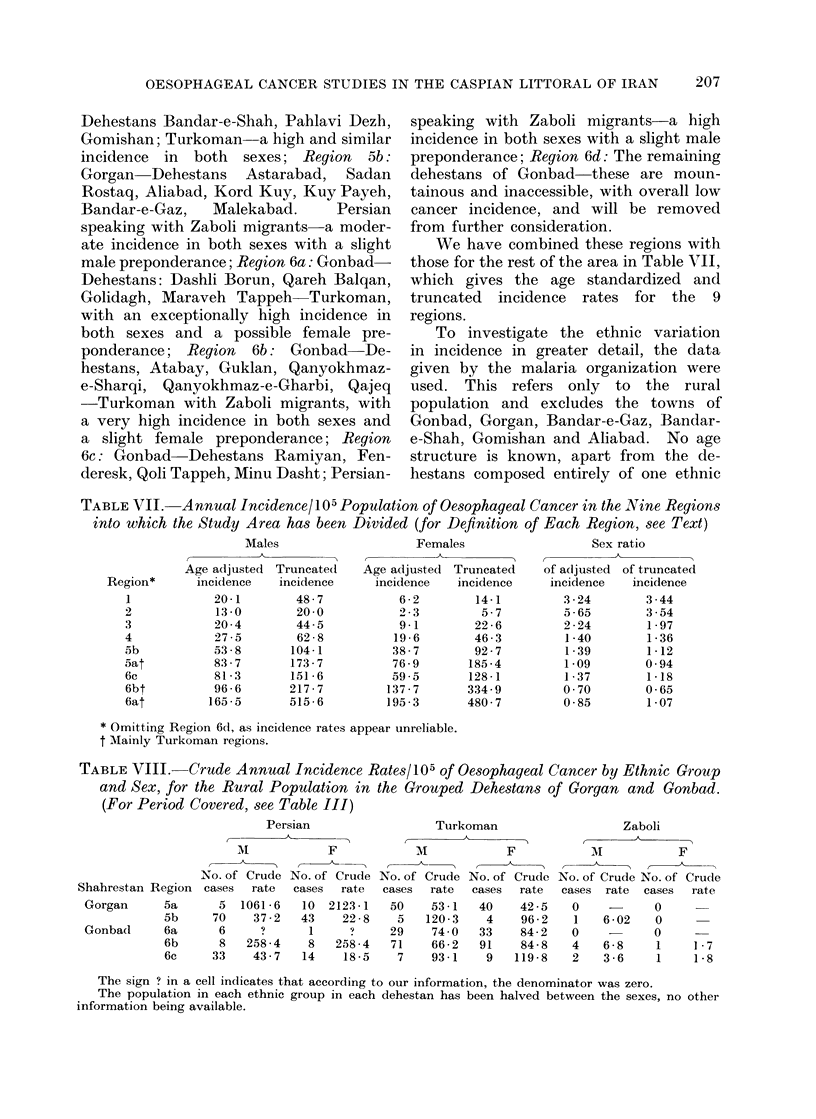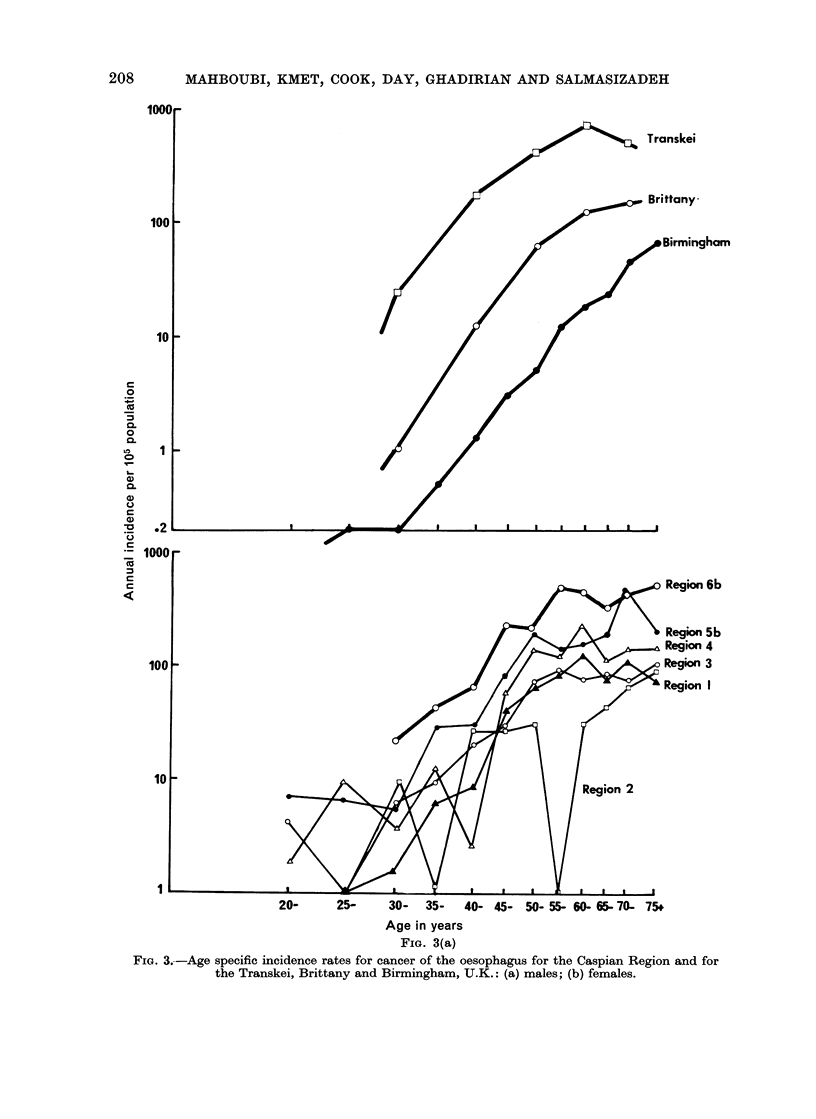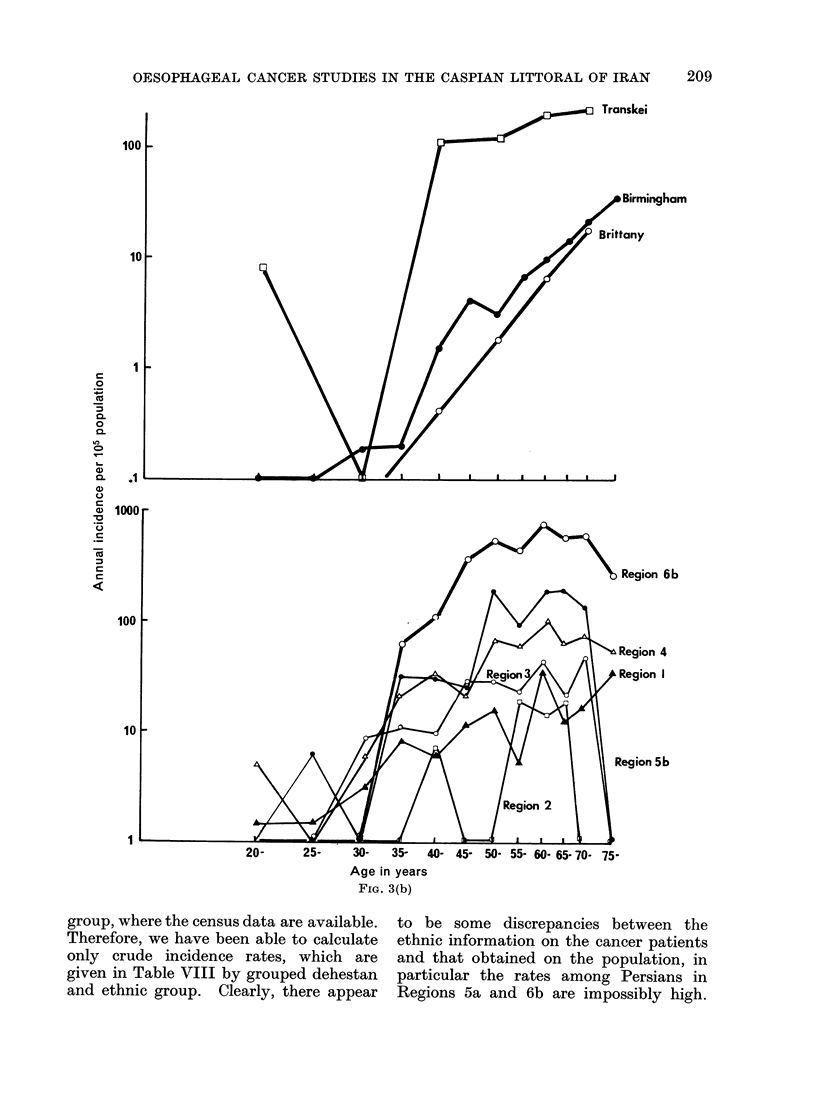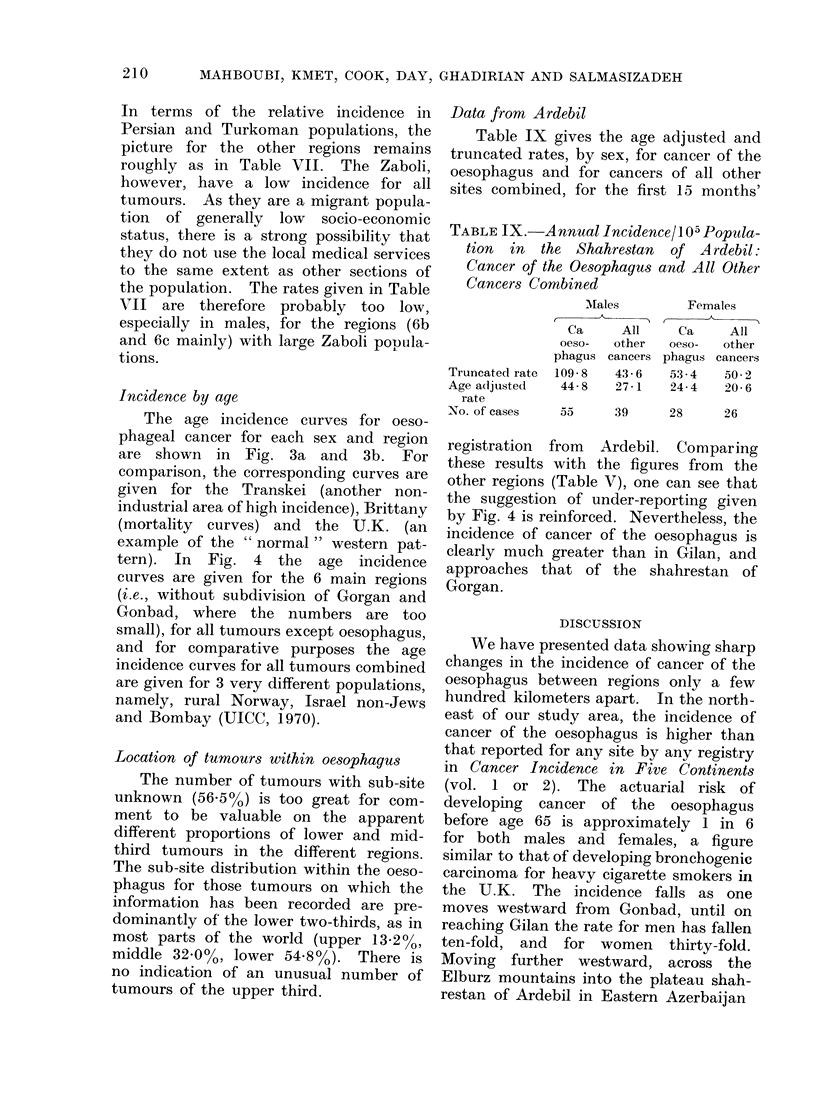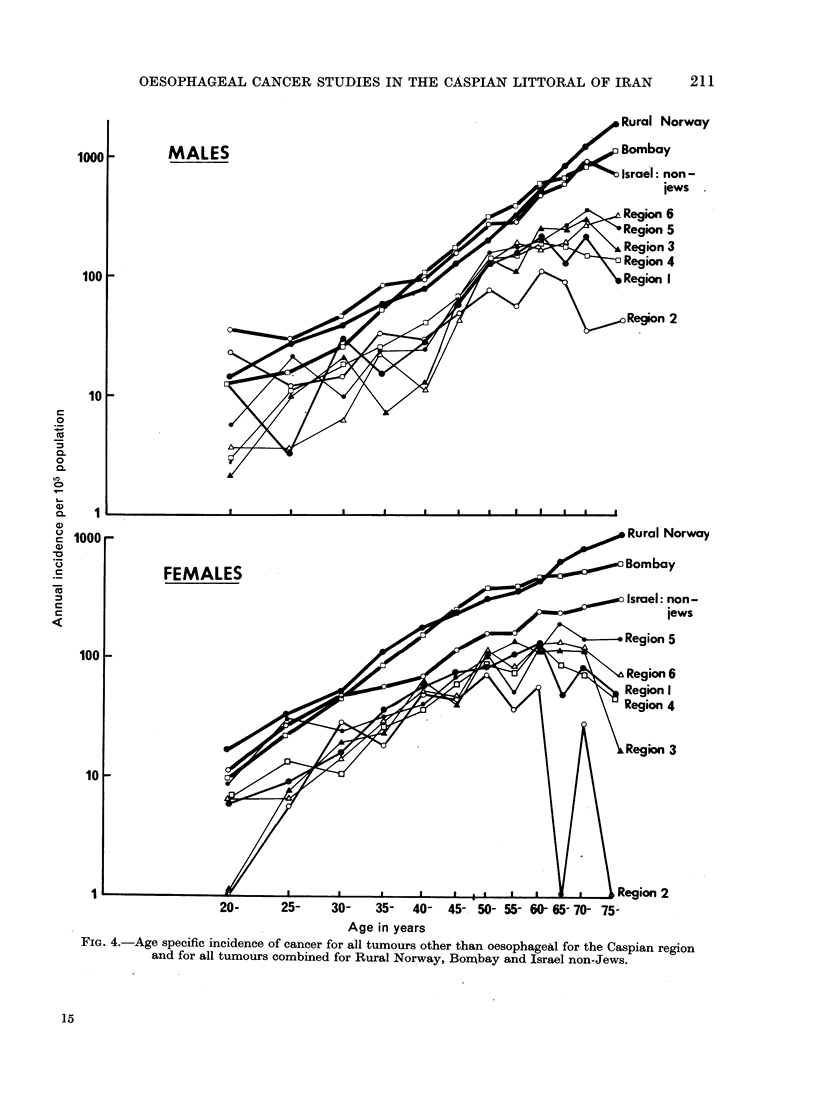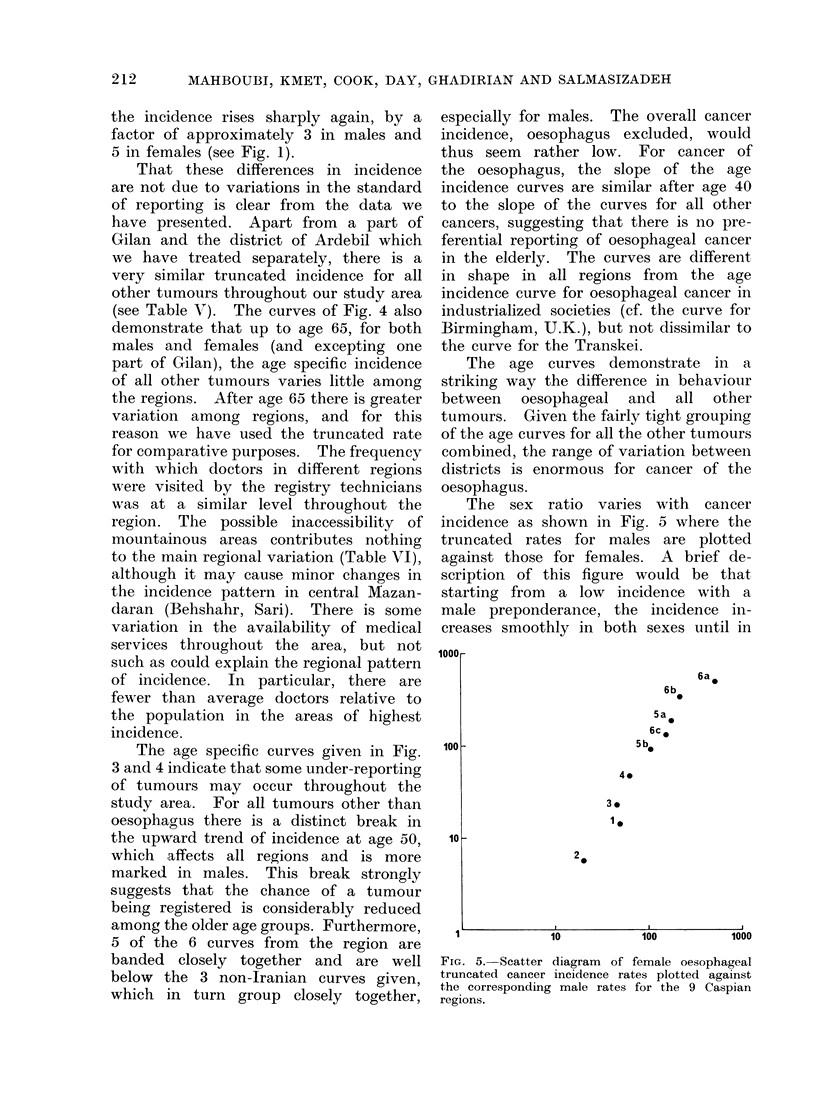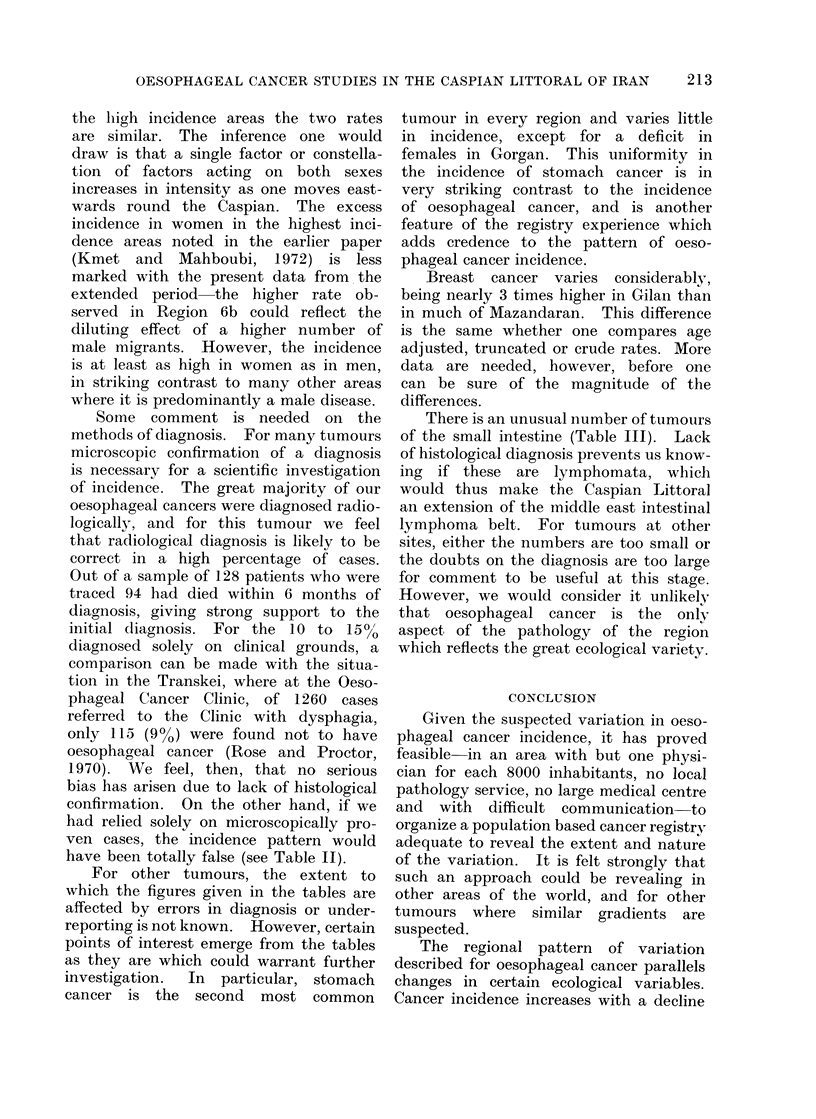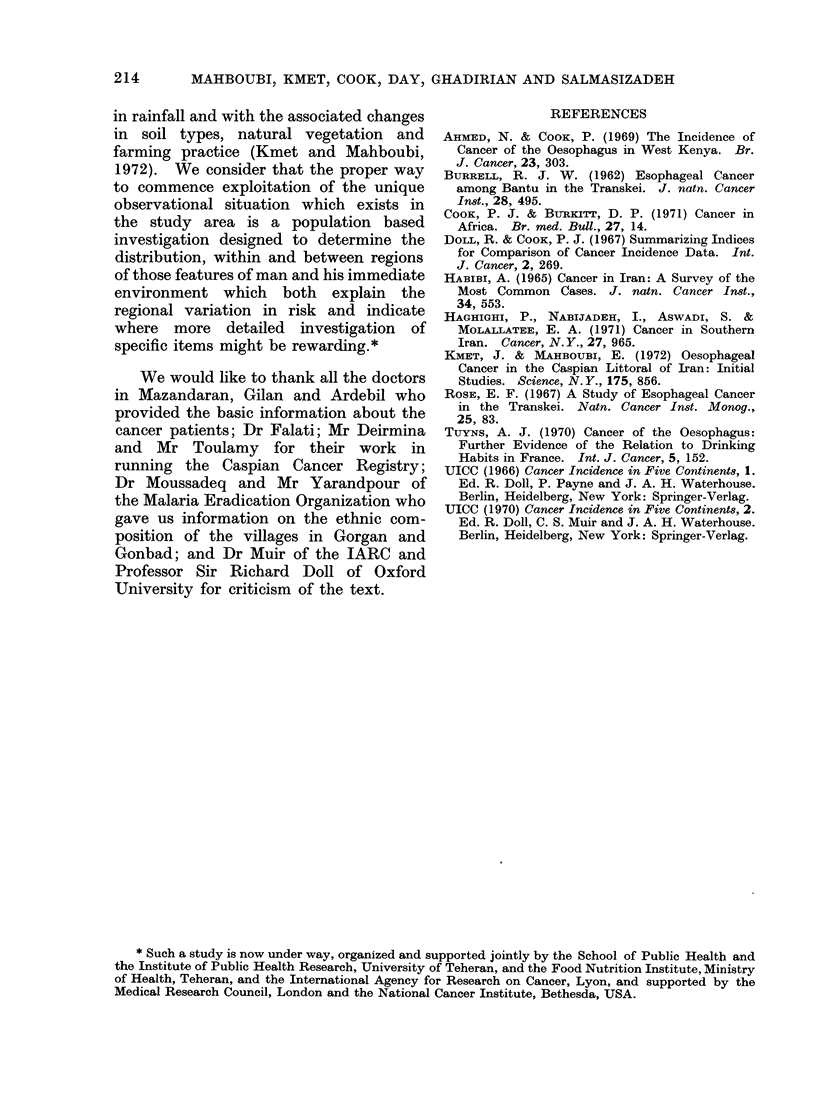# Oesophageal Cancer Studies in the Caspian Littoral of Iran: The Caspian Cancer Registry

**DOI:** 10.1038/bjc.1973.138

**Published:** 1973-09

**Authors:** E. Mahboubi, J. Kmet, P. J. Cook, N. E. Day, P. Ghadirian, S. Salmasizadeh

## Abstract

The results of the first 3 years of cancer registration on the Caspian Littoral are described. The main finding, confirming previous reports, is a very large variation within the region of the incidence of oesophageal cancer. Possible sources of bias are considered and shown to contribute little to the pattern of incidence. Among women there is a thirty-fold variation in the incidence across the regions; among men a ten-fold variation. In the north-east of the region the tumour is at least as common in women as in men, and is more common than almost any tumour anywhere in the world. Among other tumours, stomach cancer has a strikingly uniform incidence by comparison; breast cancer shows an incidence gradient of opposite slope.


Br. J. Cancer (1973) 28, 197

### OESOPHAGEAL CANCER STUDIES IN THE CASPIAN LITTORAL OF IRAN: THE CASPIAN CANCER REGISTRY

E. MAHBOUBI[1], J. KMET[2], P. J. COOK[3], N. E. DAY[2], P. GHADIRIAN[4] AND S. SALMASIZADEH[4]

*From the [1]Institute of Public Health Research, University of Teheran; [2]International Agency for Research on Cancer, Lyon, France; [3]D.H.S.S. Cancer Epidemiology and Clinical Trials Unit, Department Regius Professor Medicine, Oxford; [4]Babol Research Station, Institute of Public Health, University of Teheran*

Received 16 April 1973. Accepted 31 May 1973

**Summary.—The results of the first 3 years of cancer registration on the Caspian Littoral are described. The main finding, confirming previous reports, is a very large variation within the region of the incidence of oesophageal cancer. Possible sources of bias are considered and shown to contribute little to the pattern of incidence. Among women there is a thirty-fold variation in the incidence across the regions; among men a ten-fold variation. In the north-east of the region the tumour is at least as common in women as in men, and is more common than almost any tumour anywhere in the world. Among other tumours, stomach cancer has a strikingly uniform incidence by comparison; breast cancer shows an incidence gradient of opposite slope.**

THE Caspian Littoral supports some 4 million people in the densely populated and ecologically diverse strip of land between the Soviet border and the Caspian sea to the north and the barrier of the Elburz mountains to the south and west (Fig. 1).

During an exploratory visit to the area by one of us (J. K.) in 1966, verbal evidence was gathered suggesting large changes in the occurrence of oesophageal cancer over a relatively small area. Other reports have indicated that the disease is relatively common in Iran (Habibi, 1965; Haghighi *et al.*, 1971). These reports have been based on the records of central institutions and none attempted to establish incidence figures or regional changes in incidence. Striking gradients in the frequency of oesophageal cancer have been reported from other areas of the world (Burrell, 1962; Ahmed and Cook, 1969; Tuyns, 1970; Cook and Burkitt, 1971) and appear to be a feature of the epidemiology of the disease.

Population based studies are the methodology of choice for exploiting the differences observed in Iran, and for this purpose quantitative incidence figures were clearly necessary. Preliminary attempts to obtain more detailed information on oesophageal cancer revealed a dearth of suitable records, both in the central institutions in Teheran and in the local hospitals.

The only feasible way of obtaining adequate incidence data appeared to be the creation of a special registration scheme. The Caspian Cancer Registry was therefore established in 1969 in the town of Babol on the Caspian Littoral, by the Institute of Public Health Research, Teheran, and the International Agency for Research on Cancer. It was set up in a field research station given over for this purpose by the IPHR. The aim of the cancer registry was to establish the pattern of oesophageal cancer incidence in the area, and to investigate the incidence of other tumours. Registration was begun in the ostan (province) of Mazandaran in June 1968 and extended to Gilan Ostan in June 1969. In March 1970 registration was extended to the

FIG. 1.—Map of the Caspian Littoral, showing administrative boundaries and main topographical features of the ostans of Gilan, Mazandaran and constituent shahrestans. The shahrestan of Ardebil (Azerbaijan) is also included.

neighbouring shahrestan (district) of Ardebil to the west of Gilan, following clinical reports of a high incidence on the Iranian plateau.

An initial account of the relationship between the ecological features and the incidence of oesophageal cancer has been published (Kmet and Mahboubi, 1972), with a preliminary report of the registry results up to June 1970. The present paper gives a more extended analysis of the results to June 1971 and a description of the working of the registry.

*The organization of the Cancer Registry*

Medical facilities in the area are provided by a combination of government, charitable and private institutions—mainly in the form of small hospitals of less than 200 beds, general government clinics, private clinics offering specialist facilities such as x-ray and by numerous general practitioners in private practice. There is a total of some 500 doctors in the area; their distribution throughout the region is fairly uniform. There is thus approximately one physician for each 8000 inhabitants.

There was evidence in the areas of apparent high frequency, that the disease was sufficiently common to be popularly recognized and known to be almost invariably fatal. Under these circumstances, a villager would consult a physician and might perhaps get a second opinion or be persuaded to visit a radiologist, but once he suspected cancer of the oesophagus he might be reluctant to meet the additional expense of more extensive investigations or of palliative treatment, and might prefer to return home to die. An essential requirement of registration, therefore, was to cover the basic medical services and to include general practitioners as well as specialists. At the start of the survey a special letter, signed by the Director of the Institute of Public Health Research, was individually addressed to each doctor explaining the project and asking for co-operation. Doctors were also visited by the medical officer in charge of the cancer registry. Cancer notification forms were then distributed to all doctors in the area. To ensure maximum co-operation in the survey, the completed forms have been collected personally at regular intervals by technicians who toured the area every 4–6 weeks visiting the doctors. Checks were made regularly to ensure that doctors in the different regions were visited equally often by the technicians.

Throughout the investigation it has been stressed that doctors should report not just cancer of the oesophagus but all suspected malignant tumours, although it was clear from the outset that, whereas a fairly confident diagnosis of cancer of the oesophagus can be made by x-ray or even on clinical symptoms alone, for many other types of cancer the diagnostic facilities of the area could not guarantee reliable information.

In an attempt to improve the level of diagnosis and to encourage continued co-operation in the survey by providing a service in return for information received, the Institute of Public Health Research has established a pathology laboratory at the Babol Research Station. Medical officers working in the Registry have continued to visit doctors of the area to discuss problems of diagnosis. Lectures for local doctors have been given at the Registry by visiting specialists from Teheran.

*The notification form*

The notification form asked for the following information on each patient: (1) family name and first name of patient, (2) age, (3) sex, (4) permanent address, (5) length of residence at permanent address, (6) place of birth, (7) length of residence at place of birth, (8) ethnic group, (9) religion, (10) language, (11) occupation, (12) marital status, (13) date of first consultation, (14) diagnosis, (15) method of diagnosis, (16) speciality of doctor making diagnosis, (17) place of work of doctor making diagnosis.

The date on the notification form has been taken as the onset of the disease for the purpose of incidence studies. For all notification forms received in the Registry, special registry file cards were completed and also index cards of the patients' names, which were stored in alphabetical order to help eliminate the duplicate recording of cases. Duplications were eliminated by a thorough search of both sets of files.

*Demographic background*

The published volumes of the 1966 census of Iran provide population figures by age and sex for each shahrestan. Shahrestans are further divided into subdistricts, or dehestans. The census gives the overall population by sex of each dehestan, but no age structure. In the high incidence areas of Gorgan and Gonbad shahrestans, we felt it necessary to have a more detailed picture of the changes in cancer incidence. For this purpose a listing was obtained from the Iranian Central Statistical Office of the population by age and sex in each dehestan of Gorgan and Gonbad. The Central Statistical Office has also published a village gazetteer, based on the 1966 census, which provides extensive demographic, topographic, economic and agricultural information on all villages, *i.e.* settlements with populations less than 5000.

There has been substantial migration into some areas of the shahrestans of Gorgan and Gonbad, where there is widespread agricultural expansion. The original inhabitants were linguistically Turkoman in the north and mostly Persian-speaking in the south. The migrants are mainly from the Zabol region in the east of Iran (Zaboli). In the rest of the study area (except for Ardebil), the population is almost entirely Persian though speaking several distinct dialects. In Ardebil the language is Azeri Turkic.

The published census material has information on the number of persons born in another ostan but no ethnic details. For the cancer patients, however, the ethnic information is much more complete than that for place of birth. In order to construct the approximate ethnic composition of the general population, we therefore obtained an estimate of the ethnic composition of each village from the Malaria Eradication Organization, whose agents visit each house in every village once a month as a continuing part of the malaria surveillance. This information was available only for the rural population. We have no information on the age or sex structure of the different ethnic groups, nor on the ethnic composition of the urban population.

*The results of the Cancer Registry*

(i) *Completeness of the information obtained.*—Information on place of birth was obtained on less than 30% of the patients. Almost all cases of marriageable age were married and the great preponderance of cases claimed farming as their occupation. No further use will be made of these three variables in this paper.

For all cases registered, sex, ethnic group and ostan of residence were known. Age was given in more than 95% of cases registered, for both oesophageal and other tumours, with little geographic variation in the percentage with age unknown. In only 8 cases was shahrestan of residence unknown. Dehestan of residence was unknown, however, in 12% of oesophageal cancers and 26% of other cancers, the figures being slightly worse in Gilan than in Mazandaran.

Table I gives the number of cases recorded in each 3-monthly period of registration for all types of cancer, by broad subdivision of the area. There are no long-term trends in the level of registration, except in Ardebil, where registration was slow to get under way, making statements on incidence unreliable. This shahrestan will be treated separately for the remainder of the paper. In all other regions, however, there is considerable seasonal variation, winter

TABLE I.—*Number of Cancer Cases Registered in Each Three Month Period of Registration (Both Sexes Combined)*

| Area | Time period: 1968 July–Sept. | 1968 Oct.–Dec. | 1969 Jan.–Mar. | 1969 Apr.–June | 1969 July–Sept. | 1969 Oct.–Dec. | 1970 Jan.–Mar. | 1970 Apr.–June | 1970 July–Sept. | 1970 Oct.–Dec. | 1971 Jan.–Mar. | 1971 Apr.–June |
|---|---|---|---|---|---|---|---|---|---|---|---|---|
| Gorgan and Gonbad | 98 | 79 | 68 | 103 | 95 | 99 | 81 | 136 | 132 | 98 | 75 | 103 |
| The remainder of Mazandaran | 97 | 109 | 106 | 101 | 94 | 88 | 103 | 120 | 141 | 131 | 94 | 100 |
| Gilan | — | — | — | — | 117 | 41 | 70 | 139 | 106 | 102 | 95 | 109 |
| Ardebil | — | — | — | — | — | — | — | 22 | 11 | 31 | 35 | 49 |

TABLE II.—*Number of Cases Diagnosed by Each Method at Each Site.*

| Province | Method of diagnosis | % Oesophagus M | % Oesophagus F | % All other sites M | % All other sites F |
|---|---|---|---|---|---|
| Gilan | Clinical | 22·6 | 26·1 | 33·4 | 35·7 |
| | Radiological | 47·9 | 54·3 | 18·0 | 9·9 |
| | Cytological | 1·4 | 0·0 | 3·2 | 2·0 |
| | Histological | 28·1 | 19·6 | 41·8 | 50·0 |
| | Unknown | 0·0 | 0·0 | 3·5 | 2·4 |
| | Total no. of cases | 146 | 46 | 311 | 252 |
| Mazandaran (other than Gorgan and Gonbad) | Clinical | 17·1 | 14·5 | 27·9 | 33·5 |
| | Radiological | 65·0 | 68·8 | 25·8 | 13·2 |
| | Cytological | 0·0 | 0·0 | 3·1 | 2·0 |
| | Histological | 17·1 | 15·6 | 37·1 | 43·1 |
| | Unknown | 0·8 | 1·1 | 6·1 | 8·5 |
| | Total no. of cases | 257 | 186 | 488 | 355 |
| Gorgan | Clinical | 7·6 | 12·6 | 14·2 | 27·0 |
| | Radiological | 83·5 | 83·1 | 47·3 | 26·1 |
| | Cytological | 0·0 | 0·0 | 6·1 | 1·8 |
| | Histological | 8·8 | 5·1 | 25·7 | 39·6 |
| | Unknown | 0·0 | 0·0 | 6·1 | 5·4 |
| | Total no. of cases | 170 | 118 | 148 | 109 |
| Gonbad | Clinical | 11·6 | 16·7 | 22·4 | 36·1 |
| | Radiological | 87·0 | 82·3 | 47·7 | 38·9 |
| | Cytological | 0·0 | 0·0 | 0·0 | 1·4 |
| | Histological | 1·4 | 0·9 | 17·8 | 18·1 |
| | Unknown | 0·0 | 0·0 | 12·1 | 2·8 |
| | Total no. of cases | 216 | 215 | 107 | 74 |
| All regions (percentages) | Clinical | 14·6 | 15·8 | 25·9 | 33·6 |
| | Radiological | 71·8 | 75·8 | 27·5 | 16·3 |
| | Cytological | 0·3 | 0·0 | 7·4 | 1·9 |
| | Histological | 13·1 | 7·1 | 33·4 | 42·6 |
| | Unknown | 0·3 | 0·4 | 5·7 | 5·6 |
| | Total no. of cases | 789 | 565 | 1054 | 790 |

months generally yielding fewer cases (due probably to the difficulties in travel). Although in consequence there may be a general under-reporting of cases, we found no evidence that seasonal patterns of registration have given rise to serious regional bias.

(ii) *Method of diagnosis.*—Table II gives the method of diagnosis of oesophageal tumours and of all other tumours combined. Where more than one method of diagnosis was reported, preference was given in the following order: histological, cytological, radiological and clinical. The proportion of all diagnoses with histological confirmation is 25·6% for males and 28·2% for females. The proportion of oesophageal, stomach and lung tumours diagnosed radiologically with no histological confirmation is high, especially in Gorgan and Gonbad.

(iii) *The cancer incidence data.*—Table III gives the number of registered cases by site, sex and ostan.

Table IV gives the annual incidence rate age standardized to the world population (UICC, 1970), truncated rate (35–64 age groups) (Doll and Cook, 1967) and the crude rate, for oesophageal cancer and neoplasms of all other sites combined, by sex and shahrestan. The age adjusted and truncated rates were adjusted to take account of cases with age unknown.

As the number of cases per shahrestan is often rather small, further analysis is based on groupings of shahrestans. The grouping has been made on the basis of the oesophageal cancer incidence and geographical proximity. There is an arbitrary element in any grouping based on data of the type given in Table IV. However, there is clearly a group of shahrestans in Gilan with a lower incidence, and we have taken as this group the three contiguous shahrestans of Fowman, Sowma-Ehsara and Rudbar, which alone show a lower incidence in both sexes. In Mazandaran Ostan, Amol and Shahi have higher incidence than Babol and Nowshahr. We have grouped Sari, Behshahr and Nur with Amol and Shahi, and Shahsavar with Babol and Nowshahr, partly on the pattern of incidence in the 2 sexes, partly on geographic proximity and partly because the figures from Behshahr and Sari appear affected by under-reporting from mountainous areas, and may therefore have incidence levels close to those in Amol and Shahi. Both Gorgan and Gonbad, with a large number of cases, are clearly distinct. Our regions are thus defined as follows: *Group* 1: Gilan—Shahrestans Astara, Tavalesh, Lahijan, Langarud, Bandar-Pahlavi, Rasht and Rudsar; a low incidence in men and a very low incidence in women; *Group* 2: Gilan—Shahrestans Rudbar, Fowman, Sowma-Ehsara; a very low incidence in both sexes; *Group* 3: Mazandaran—Shahrestans Shahsavar, Nowshahr, Babol; a low incidence in men and low to very low in women; *Group* 4: Mazandaran—Shahrestans Nur, Amol, Shahi, Sari, Behshahr; a moderate incidence in men and a low incidence in women; *Group* 5: Mazandaran—Gorgan Shahrestan; a high incidence in both sexes; *Group* 6: Mazandaran—Gonbad Shahrestan; a very high incidence in both sexes, with a possible female preponderance.

This grouping gives regions which are geographically continuous except that Babol shahrestan is taken from the centre of Group 4 and included with the non-adjacent shahrestans of Group 3.

Table V gives, for these grouped shahrestans, the age standardized truncated incidence of cancers of the oesophagus, stomach, skin, breast, cervix, lung, larynx, tongue, colon, rectum and liver, and of all tumours other than oesophagus.

On the basis of the figures for all sites other than the oesophagus, there appears to be some under-reporting from Region 2, compared with the other regions.

As parts of Region 2 are very mountainous, we investigated the possibility that mountainous areas, because of their inaccessibility, report fewer tumours than other areas. We classified each dehestan in each shahrestan as being mountainous, part mountainous–part plain, or plain by the proportion of villages in each dehestan classified as plain or mountainous in the Village Gazetteers. The crude incidence rates for oesophageal cancer and cancer of all other sites are given in Table VI by grouped shahrestan for each topographical category. One can see a tendency for mountainous areas to have lower rates, but it is not uniform and

TABLE III.—*Numbers of Cancers Recorded at Each Site, by Sex and Ostan: Mazandaran: July* 1968–*June* 1971; *Gilan: July* 1969–*June* 1971; *Shahrestan of Ardebil: April* 1970–*June* 1971 (*Ostan of Eastern Azerbaijan*)

| Site ICD 8th revision | Mazandaran | | Gilan | | Ardebil | | Total | | |
|---|---|---|---|---|---|---|---|---|---|
| | M | F | M | F | M | F | M | F | Both |
| 140 Lip | 14 | 2 | 7 | 3 | 3 | — | 25 | 5 | 30 |
| 141 Tongue | 6 | 23 | 13 | 6 | — | 1 | 19 | 30 | 49 |
| 142 Salivary gland | 2 | 2 | — | — | — | 2 | 2 | 4 | 4 |
| 143 Gum | 2 | 4 | 4 | 1 | — | — | 6 | 3 | 9 |
| 144 Floor of mouth | — | — | — | — | — | — | — | — | — |
| 145 Other mouth | 2 | 2 | 2 | 2 | — | — | 5 | 8 | 13 |
| 146 Oropharynx | 4 | 1 | 1 | — | — | 1 | 5 | 2 | 7 |
| 147 Nasopharynx | — | — | — | — | — | — | — | — | — |
| 148 Hypopharynx | 5 | 3 | 7 | 5 | — | — | 12 | 8 | 20 |
| 149 Pharynx | 8 | 3 | 1 | — | — | — | 9 | 3 | 12 |
| 150 Oesophagus | 643 | 519 | 146 | 46 | 55 | 28 | 844 | 593 | 1437 |
| 151 Stomach | 222 | 97 | 67 | 31 | 8 | 4 | 297 | 132 | 429 |
| 152 Small intestine | 16 | 2 | 7 | 3 | — | 1 | 23 | 6 | 29 |
| 153 Colon | 22 | 10 | 11 | 5 | — | 2 | 32 | 18 | 50 |
| 154 Rectum | 13 | 11 | 4 | 5 | 2 | — | 19 | 16 | 35 |
| 155 Liver | 41 | 22 | 12 | 5 | 3 | 2 | 55 | 27 | 82 |
| 156 Gall bladder and bile duct | 2 | — | — | — | — | — | 2 | — | 2 |
| 157 Pancreas | 14 | 4 | 1 | — | — | — | 15 | 4 | 19 |
| 158 Peritoneum | 3 | 6 | 1 | 2 | — | — | 4 | 8 | 12 |
| 159 Digestive tract, unspecified | 5 | 6 | — | 1 | — | — | 5 | 7 | 12 |
| 161 Larynx | 51 | 4 | 34 | 6 | 4 | 1 | 89 | 11 | 100 |
| 162 Lung, bronchus | 66 | 22 | 16 | 2 | 5 | 1 | 87 | 25 | 112 |
| 163 Unspecified resp. | 6 | 3 | — | — | — | — | 6 | 3 | 3 |
| 170 Bone | 15 | 14 | 8 | 8 | — | 2 | 23 | 24 | 47 |
| 171 Connective tissue | 12 | 13 | 6 | 7 | — | — | 19 | 21 | 39 |
| 172 Haemangioma | 3 | 6 | 4 | 1 | — | — | 7 | 7 | 14 |
| 173 Other skin | 58 | 58 | 38 | 30 | 3 | 1 | 99 | 89 | 188 |
| 174 Breast | 1 | 54 | 2 | 45 | — | 1 | 3 | 100 | 103 |
| 180 Cervix | — | 54 | — | 34 | — | 1 | — | 89 | 89 |
| 181 Corionepithelioma | — | 4 | — | 1 | — | — | — | 5 | 5 |
| 182 Uterus | — | 10 | — | 1 | — | — | — | 11 | 11 |
| 183 Ovary | — | 14 | — | 6 | — | — | — | 21 | 21 |
| 184 Other f. gen. | — | 6 | — | 1 | — | — | — | 7 | 7 |
| 185 Prostate | 5 | — | 1 | — | — | — | 6 | — | 6 |
| 186 Testis | 15 | — | 12 | — | — | — | 27 | — | 27 |
| 187 Other male gen. | 1 | — | — | — | — | — | 1 | — | 1 |
| 188 Bladder | 21 | 5 | 8 | 3 | — | — | 29 | 8 | 37 |
| 189 Other urinary/ kidney | 7 | 4 | 3 | — | — | — | 10 | 4 | 14 |
| 190 Eye | 8 | 4 | 3 | 5 | — | — | 11 | 9 | 20 |
| 191 Brain | 2 | — | 3 | — | — | — | 5 | — | 5 |
| 192 Other nervous system | — | — | 1 | — | — | — | 1 | — | 1 |
| 193 Thyroid | 6 | 6 | 3 | 3 | 1 | 1 | 10 | 10 | 20 |
| 194 Endocrine | 1 | — | — | — | — | — | 1 | — | 1 |
| 196 Lymph nodes secondary | 6 | 3 | 2 | 3 | — | — | 8 | 6 | 14 |
| 197 Secondary lung and digestive | 10 | 8 | 2 | 2 | — | — | 13 | 11 | 24 |
| 198 Other secondary | 1 | — | — | — | — | — | 1 | — | 1 |
| 199 Unspecified sites | 4 | 15 | — | 3 | 1 | 2 | 6 | 17 | 23 |
| 200 and 202 Lymphosarcoma and reticulum cell sarcoma | 26 | 13 | 13 | 12 | 2 | 1 | 41 | 26 | 67 |
| 201 Hodgkin's disease | 14 | 9 | 7 | 5 | 4 | 0 | 25 | 14 | 39 |
| 203 Multiple myeloma | 1 | — | — | — | — | — | 1 | — | 1 |
| 204 Lymphatic leukaemia | 11 | 6 | 3 | 2 | 3 | 2 | 17 | 10 | 27 |
| 205 Leukaemia, myeloid | 5 | 1 | — | 2 | — | — | 5 | 3 | 8 |
| 207 Other leukaemias | 8 | 6 | 4 | 1 | — | — | 12 | 7 | 19 |
| TOTAL | 1386 | 1057 | 457 | 298 | 94 | 54 | 1937 | 1409 | 3346 |

TABLE IV.—*The Annual Incidence*/$10^5$ Population of Oesophageal Tumours, and of all Other Tumours, in the Shahrestans of Mazandaran and Gilan*

| | | Oesophageal cancer | | | | | | | All other cancers | | | | | | |
|---|---|---|---|---|---|---|---|---|---|---|---|---|---|---|---|
| | | Males | | | Females | | | % Age unknown | Males | | | Females | | | % Age unknown |
| Ostan | Shahrestan | Age* adj. | Truncated | Crude | Age* adj. | Truncated | Crude | both sexes | Age* adj. | Truncated | Crude | Age* adj. | Truncated | Crude | both sexes |
| Mazandaran | Gonbad | 93·1 | 206·4 | 48·3 | 110·0 | 262·9 | 52·8 | 1·9 | 47·7 | 83·4 | 23·9 | 33·3 | 68·8 | 18·2 | 5·6 |
| | Gorgan | 66·7 | 123·8 | 35·3 | 49·2 | 110·1 | 26·4 | 6·3 | 56·8 | 96·6 | 30·7 | 41·9 | 67·4 | 24·8 | 6·6 |
| | Behshahr | 26·0 | 48·9 | 13·9 | 20·0 | 40·1 | 12·6 | 4·4 | 60·9 | 125·2 | 33·5 | 44·1 | 78·3 | 27·0 | 8·7 |
| | Sari | 17·6 | 46·7 | 9·9 | 16·8 | 29·3 | 9·6 | 3·3 | 37·5 | 78·0 | 22·3 | 19·7 | 42·9 | 13·3 | 2·8 |
| | Shahi | 32·5 | 59·3 | 17·1 | 25·5 | 62·9 | 14·7 | 8·2 | 45·8 | 76·2 | 26·1 | 24·0 | 53·5 | 14·0 | 5·8 |
| | Babol | 24·0 | 42·5 | 12·4 | 8·6 | 19·8 | 7·9 | 2·8 | 47·1 | 75·8 | 33·5 | 32·8 | 74·0 | 20·0 | 1·1 |
| | Amol | 37·7 | 91·9 | 18·8 | 23·6 | 55·6 | 14·6 | 3·5 | 47·9 | 108·7 | 26·9 | 39·3 | 96·8 | 22·4 | 6·3 |
| | Nur | 35·9 | 90·3 | 20·0 | 12·8 | 27·5 | 8·3 | 10·5 | 23·7 | 52·6 | 15·7 | 26·9 | 46·2 | 19·4 | 0·0 |
| | Nowshahr | 14·9 | 40·9 | 8·9 | 12·7 | 27·2 | 7·4 | 3·4 | 33·6 | 63·0 | 18·9 | 22·4 | 31·8 | 14·2 | 9·4 |
| | Shahsavar | 19·9 | 51·9 | 16·1 | 10·4 | 23·5 | 6·4 | 5·9 | 58·9 | 120·3 | 30·2 | 48·7 | 113·2 | 28·7 | 4·2 |
| Gilan | Rudsar | 28·9 | 56·0 | 17·4 | 6·2 | 8·0 | 3·7 | 6·9 | 36·3 | 78·7 | 23·2 | 23·1 | 78·8 | 20·8 | 6·6 |
| | Langarud | 12·1 | 24·7 | 7·4 | 16·5 | 44·0 | 10·3 | 0·0 | 62·1 | 144·2 | 32·4 | 31·8 | 64·2 | 23·6 | 2·6 |
| | Lahijan | 21·3 | 51·4 | 11·4 | 4·9 | 12·8 | 3·5 | 2·9 | 24·1 | 38·0 | 12·7 | 27·4 | 52·7 | 17·0 | 2·9 |
| | Rasht | 21·7 | 46·1 | 12·2 | 4·1 | 7·6 | 2·6 | 9·3 | 47·1 | 98·6 | 31·9 | 33·5 | 80·6 | 23·4 | 5·6 |
| | Bandar-Pahlavi | 34·0 | 39·9 | 16·5 | 12·8 | 32·1 | 8·5 | 20·0 | 64·1 | 121·7 | 43·0 | 33·4 | 91·8 | 25·4 | 0·0 |
| | Rudbar | 11·6 | 22·3 | 6·5 | 3·0 | 9·8 | 1·4 | 16·7 | 13·9 | 23·2 | 10·5 | 10·6 | 21·3 | 8·3 | 0·0 |
| | Sowma-Ehsara | 16·5 | 17·1 | 7·7 | 12·0 | 4·0 | 1·1 | 0·0 | 36·5 | 68·1 | 20·1 | 22·5 | 62·3 | 15·1 | 6·1 |
| | Fowman | 10·4 | 21·0 | 7·3 | 2·7 | 4·4 | 1·6 | 0·0 | 28·8 | 65·8 | 18·5 | 21·1 | 48·1 | 13·9 | 5·0 |
| | Tavalesh | 22·7 | 57·0 | 10·5 | 9·8 | 15·3 | 5·9 | 0·0 | 44·3 | 102·5 | 21·8 | 40·9 | 106·1 | 22·0 | 5·5 |
| | Astara | 19·6 | 63·2 | 10·5 | 7·1 | 23·0 | 3·6 | 0·0 | 72·2 | 146·2 | 34·8 | 48·7 | 88·8 | 25·5 | 0·0 |

TABLE V.—*Annual Truncated Incidence/$10^5$ Population of Certain Tumours in Grouped Shahrestans of Gilan and Mazandaran (ICD 8th Revision)*

Males

| | Tumour site | | | | | | | | |
|---|---|---|---|---|---|---|---|---|---|
| Region* | Oesophagus 150 | All sites other than oesophagus | Tongue 141 | Stomach 151 | Colon and rectum 153 and 154 | Liver 155 | Larynx 161 | Lung 162 | Skin 173 |
| 1 | 48·7 | 87·6 | 3·8 | 28·3 | 4·7 | 2·9 | 11·7 | 5·4 | 8·2 |
| 2 | 20·0 | 54·7 | 3·9 | 8·0 | 2·2 | 6·2 | 4·7 | 6·2 | 5·1 |
| 3 | 44·5 | 83·5 | 2·8 | 26·9 | 1·4 | 1·3 | 7·9 | 4·1 | 8·1 |
| 4 | 62·7 | 89·9 | 0·7 | 35·1 | 3·9 | 4·4 | 7·1 | 11·1 | 9·8 |
| 5 | 123·9 | 96·1 | — | 31·7 | 5·5 | 6·1 | 9·7 | 8·4 | 8·6 |
| 6 | 206·4 | 83·4 | — | 32·7 | 1·6 | 5·7 | 9·5 | 3·5 | 6·0 |

Females

| | Tumour site | | | | | | | | | | |
|---|---|---|---|---|---|---|---|---|---|---|---|
| Region | Oesophagus 150 | All sites other than oesophagus | Tongue 141 | Stomach 151 | Colon and rectum 153 and 154 | Liver 155 | Larynx 161 | Lung 162 | Skin 173 | Breast 174 | Cervix 180 |
| 1 | 14·1 | 76·4 | 1·6 | 11·2 | 1·4 | 1·1 | 1·9 | 1·3 | 10·3 | 17·2 | 12·0 |
| 2 | 5·6 | 45·7 | — | 3·0 | 1·4 | 1·9 | — | — | 4·5 | 10·2 | 9·1 |
| 3 | 22·6 | 74·5 | 7·5 | 14·8 | 2·3 | 2·0 | — | 0·7 | 10·7 | 7·0 | 7·7 |
| 4 | 46·3 | 63·3 | 0·6 | 16·8 | 2·5 | 1·0 | — | 3·0 | 7·1 | 8·1 | 6·2 |
| 5 | 110·1 | 67·4 | 3·1 | 6·6 | 4·9 | 6·3 | 1·2 | 4·6 | 4·4 | 10·0 | 2·7 |
| 6 | 262·9 | 68·8 | — | 16·9 | 1·6 | 4·9 | — | 3·3 | 3·3 | 6·4 | 12·1 |

* For definition see text and Fig. 1.

TABLE VI.—*Crude Annual Incidence Rates/$10^5$ Population of Cancer of the Oesophagus and of All Other Tumours for the Main Topographical Divisions of Each Region*

| | | Population | | | Crude incidence rates: Oesophageal cancer | | | Crude incidence rates: All other cancers | | |
|---|---|---|---|---|---|---|---|---|---|---|
| Region | Sex | Mountain | Part mountain/part plain | Plain | Mountain | Part mountain/part plain | Plain | Mountain | Part mountain/part plain | Plain |
| 1 | Male | 26,699 | 113,106 | 231,125 | 7·49 | 6·63 | 8·87 | 13·11 | 17·24 | 14·28 |
| | Female | 25,584 | 109,208 | 233,451 | 1·95 | 2·29 | 4·07 | 13·68 | 14·65 | 8·99 |
| 2 | Male | 20,045 | 37,008 | 77,658 | 4·98 | 8·10 | 6·43 | 4·98 | 4·05 | 10·95 |
| | Female | 18,954 | 34,910 | 78,958 | — | — | 0·63 | 2·64 | 4·30 | 8·23 |
| 3 | Male | 25,798 | 73,576 | 120,899 | 7·75 | 11·33 | 9·93 | 10·34 | 24·91 | 20·95 |
| | Female | 25,367 | 72,620 | 117,659 | 1·31 | 9·18 | 4·81 | 13·14 | 24·22 | 13·89 |
| 4 | Male | 58,977 | 30,434 | 184,164 | 9·60 | 8·76 | 13·21 | 13·57 | 12·05 | 18·82 |
| | Female | 48,817 | 29,401 | 181,783 | 7·36 | 9·06 | 11·73 | 6·80 | 6·81 | 11·00 |
| 5 | Male | 1,696 | 19,106 | 111,799 | 19·7 | 45·4 | 32·8 | 78·8 | 44·6 | 24·7 |
| | Female | 1,918 | 17,532 | 103,433 | 69·5 | 17·1 | 28·4 | 34·7 | 26·6 | 20·9 |
| 6 | Male | 16,727 | 9,747 | 101,046 | 19·9 | 71·8 | 48·5 | 4·0 | 13·3 | 13·8 |
| | Female | 15,226 | 8,598 | 92,839 | 28·5 | 50·4 | 54·2 | 4·3 | 12·1 | 13·3 |

Discrepancies between this table and Table VII are due to cases for which dehestan of residence is unknown.

could explain only very little of the observed regional differences in incidence of cancer of the oesophagus.

In the 2 shahrestans of Gorgan and Gonbad, where there have been sufficient cases to make a finer subdivision worthwhile, the oesophageal cancer incidence was analysed by dehestan and main ethnic group. A map of the region showing the administrative boundaries is given in Fig. 2. As the number of cases from each dehestan is often small, we have grouped dehestans on the basis of the incidence, the ethnic and topographic information and on geographic proximity (see Fig. 2) as follows: *Region 5a*: Gorgan—

FIG. 2.—Map of the Shahrestans of Gorgan and Gonbad, showing administrative boundaries of the constituent dehestans.

Dehestans Bandar-e-Shah, Pahlavi Dezh, Gomishan; Turkoman—a high and similar incidence in both sexes; *Region 5b:* Gorgan—Dehestans Astarabad, Sadan Rostaq, Aliabad, Kord Kuy, Kuy Payeh, Bandar-e-Gaz, Malekabad. Persian speaking with Zaboli migrants—a moderate incidence in both sexes with a slight male preponderance; *Region 6a:* Gonbad—Dehestans: Dashli Borun, Qareh Balqan, Golidagh, Maraveh Tappeh—Turkoman, with an exceptionally high incidence in both sexes and a possible female preponderance; *Region 6b:* Gonbad—Dehestans, Atabay, Guklan, Qanyokhmaz-e-Sharqi, Qanyokhmaz-e-Gharbi, Qajeq —Turkoman with Zaboli migrants, with a very high incidence in both sexes and a slight female preponderance; *Region 6c:* Gonbad—Dehestans Ramiyan, Fenderesk, Qoli Tappeh, Minu Dasht; Persian-speaking with Zaboli migrants—a high incidence in both sexes with a slight male preponderance; *Region 6d:* The remaining dehestans of Gonbad—these are mountainous and inaccessible, with overall low cancer incidence, and will be removed from further consideration.

We have combined these regions with those for the rest of the area in Table VII, which gives the age standardized and truncated incidence rates for the 9 regions.

To investigate the ethnic variation in incidence in greater detail, the data given by the malaria organization were used. This refers only to the rural population and excludes the towns of Gonbad, Gorgan, Bandar-e-Gaz, Bandar-e-Shah, Gomishan and Aliabad. No age structure is known, apart from the dehestans composed entirely of one ethnic

TABLE VII.—*Annual Incidence/$10^5$ Population of Oesophageal Cancer in the Nine Regions into which the Study Area has been Divided (for Definition of Each Region, see Text)*

| | Males | | Females | | Sex ratio | |
|---|---|---|---|---|---|---|
| Region* | Age adjusted incidence | Truncated incidence | Age adjusted incidence | Truncated incidence | of adjusted incidence | of truncated incidence |
| 1 | 20·1 | 48·7 | 6·2 | 14·1 | 3·24 | 3·44 |
| 2 | 13·0 | 20·0 | 2·3 | 5·7 | 5·65 | 3·54 |
| 3 | 20·4 | 44·5 | 9·1 | 22·6 | 2·24 | 1·97 |
| 4 | 27·5 | 62·8 | 19·6 | 46·3 | 1·40 | 1·36 |
| 5b | 53·8 | 104·1 | 38·7 | 92·7 | 1·39 | 1·12 |
| 5a† | 83·7 | 173·7 | 76·9 | 185·4 | 1·09 | 0·94 |
| 6c | 81·3 | 151·6 | 59·5 | 128·1 | 1·37 | 1·18 |
| 6b† | 96·6 | 217·7 | 137·7 | 334·9 | 0·70 | 0·65 |
| 6a† | 165·5 | 515·6 | 195·3 | 480·7 | 0·85 | 1·07 |

* Omitting Region 6d, as incidence rates appear unreliable.
† Mainly Turkoman regions.

TABLE VIII.—*Crude Annual Incidence Rates/$10^5$ of Oesophageal Cancer by Ethnic Group and Sex, for the Rural Population in the Grouped Dehestans of Gorgan and Gonbad. (For Period Covered, see Table III)*

| | | Persian | | | | Turkoman | | | | Zaboli | | | |
|---|---|---|---|---|---|---|---|---|---|---|---|---|---|
| | | M | | F | | M | | F | | M | | F | |
| Shahrestan | Region | No. of cases | Crude rate | No. of cases | Crude rate | No. of cases | Crude rate | No. of cases | Crude rate | No. of cases | Crude rate | No. of cases | Crude rate |
| Gorgan | 5a | 5 | 1061·6 | 10 | 2123·1 | 50 | 53·1 | 40 | 42·5 | 0 | — | 0 | — |
| | 5b | 70 | 37·2 | 43 | 22·8 | 5 | 120·3 | 4 | 96·2 | 1 | 6·02 | 0 | — |
| Gonbad | 6a | 6 | ? | 1 | ? | 29 | 74·0 | 33 | 84·2 | 0 | — | 0 | — |
| | 6b | 8 | 258·4 | 8 | 258·4 | 71 | 66·2 | 91 | 84·8 | 4 | 6·8 | 1 | 1·7 |
| | 6c | 33 | 43·7 | 14 | 18·5 | 7 | 93·1 | 9 | 119·8 | 2 | 3·6 | 1 | 1·8 |

The sign ? in a cell indicates that according to our information, the denominator was zero.

The population in each ethnic group in each dehestan has been halved between the sexes, no other information being available.

FIG. 3(a)

FIG. 3.—Age specific incidence rates for cancer of the oesophagus for the Caspian Region and for the Transkei, Brittany and Birmingham, U.K.: (a) males; (b) females.

FIG. 3(b)

group, where the census data are available. Therefore, we have been able to calculate only crude incidence rates, which are given in Table VIII by grouped dehestan and ethnic group. Clearly, there appear to be some discrepancies between the ethnic information on the cancer patients and that obtained on the population, in particular the rates among Persians in Regions 5a and 6b are impossibly high.

In terms of the relative incidence in Persian and Turkoman populations, the picture for the other regions remains roughly as in Table VII. The Zaboli, however, have a low incidence for all tumours. As they are a migrant population of generally low socio-economic status, there is a strong possibility that they do not use the local medical services to the same extent as other sections of the population. The rates given in Table VII are therefore probably too low, especially in males, for the regions (6b and 6c mainly) with large Zaboli populations.

*Incidence by age*

The age incidence curves for oesophageal cancer for each sex and region are shown in Fig. 3a and 3b. For comparison, the corresponding curves are given for the Transkei (another non-industrial area of high incidence), Brittany (mortality curves) and the U.K. (an example of the " normal " western pattern). In Fig. 4 the age incidence curves are given for the 6 main regions (*i.e.*, without subdivision of Gorgan and Gonbad, where the numbers are too small), for all tumours except oesophagus, and for comparative purposes the age incidence curves for all tumours combined are given for 3 very different populations, namely, rural Norway, Israel non-Jews and Bombay (UICC, 1970).

*Location of tumours within oesophagus*

The number of tumours with sub-site unknown (56·5%) is too great for comment to be valuable on the apparent different proportions of lower and mid-third tumours in the different regions. The sub-site distribution within the oesophagus for those tumours on which the information has been recorded are predominantly of the lower two-thirds, as in most parts of the world (upper 13·2%, middle 32·0%, lower 54·8%). There is no indication of an unusual number of tumours of the upper third.

*Data from Ardebil*

Table IX gives the age adjusted and truncated rates, by sex, for cancer of the oesophagus and for cancers of all other sites combined, for the first 15 months' registration from Ardebil. Comparing these results with the figures from the other regions (Table V), one can see that the suggestion of under-reporting given by Fig. 4 is reinforced. Nevertheless, the incidence of cancer of the oesophagus is clearly much greater than in Gilan, and approaches that of the shahrestan of Gorgan.

TABLE IX.—*Annual Incidence/$10^5$ Population in the Shahrestan of Ardebil: Cancer of the Oesophagus and All Other Cancers Combined*

| | Males | | Females | |
|---|---|---|---|---|
| | Ca oesophagus | All other cancers | Ca oesophagus | All other cancers |
| Truncated rate | 109·8 | 43·6 | 53·4 | 50·2 |
| Age adjusted rate | 44·8 | 27·1 | 24·4 | 20·6 |
| No. of cases | 55 | 39 | 28 | 26 |

DISCUSSION

We have presented data showing sharp changes in the incidence of cancer of the oesophagus between regions only a few hundred kilometers apart. In the north-east of our study area, the incidence of cancer of the oesophagus is higher than that reported for any site by any registry in *Cancer Incidence in Five Continents* (vol. 1 or 2). The actuarial risk of developing cancer of the oesophagus before age 65 is approximately 1 in 6 for both males and females, a figure similar to that of developing bronchogenic carcinoma for heavy cigarette smokers in the U.K. The incidence falls as one moves westward from Gonbad, until on reaching Gilan the rate for men has fallen ten-fold, and for women thirty-fold. Moving further westward, across the Elburz mountains into the plateau shahrestan of Ardebil in Eastern Azerbaijan

FIG. 4.—Age specific incidence of cancer for all tumours other than oesophageal for the Caspian region and for all tumours combined for Rural Norway, Bombay and Israel non-Jews.

the incidence rises sharply again, by a factor of approximately 3 in males and 5 in females (see Fig. 1).

That these differences in incidence are not due to variations in the standard of reporting is clear from the data we have presented. Apart from a part of Gilan and the district of Ardebil which we have treated separately, there is a very similar truncated incidence for all other tumours throughout our study area (see Table V). The curves of Fig. 4 also demonstrate that up to age 65, for both males and females (and excepting one part of Gilan), the age specific incidence of all other tumours varies little among the regions. After age 65 there is greater variation among regions, and for this reason we have used the truncated rate for comparative purposes. The frequency with which doctors in different regions were visited by the registry technicians was at a similar level throughout the region. The possible inaccessibility of mountainous areas contributes nothing to the main regional variation (Table VI), although it may cause minor changes in the incidence pattern in central Mazandaran (Behshahr, Sari). There is some variation in the availability of medical services throughout the area, but not such as could explain the regional pattern of incidence. In particular, there are fewer than average doctors relative to the population in the areas of highest incidence.

The age specific curves given in Fig. 3 and 4 indicate that some under-reporting of tumours may occur throughout the study area. For all tumours other than oesophagus there is a distinct break in the upward trend of incidence at age 50, which affects all regions and is more marked in males. This break strongly suggests that the chance of a tumour being registered is considerably reduced among the older age groups. Furthermore, 5 of the 6 curves from the region are banded closely together and are well below the 3 non-Iranian curves given, which in turn group closely together, especially for males. The overall cancer incidence, oesophagus excluded, would thus seem rather low. For cancer of the oesophagus, the slope of the age incidence curves are similar after age 40 to the slope of the curves for all other cancers, suggesting that there is no preferential reporting of oesophageal cancer in the elderly. The curves are different in shape in all regions from the age incidence curve for oesophageal cancer in industrialized societies (cf. the curve for Birmingham, U.K.), but not dissimilar to the curve for the Transkei.

The age curves demonstrate in a striking way the difference in behaviour between oesophageal and all other tumours. Given the fairly tight grouping of the age curves for all the other tumours combined, the range of variation between districts is enormous for cancer of the oesophagus.

The sex ratio varies with cancer incidence as shown in Fig. 5 where the truncated rates for males are plotted against those for females. A brief description of this figure would be that starting from a low incidence with a male preponderance, the incidence increases smoothly in both sexes until in

FIG. 5.—Scatter diagram of female oesophageal truncated cancer incidence rates plotted against the corresponding male rates for the 9 Caspian regions.

the high incidence areas the two rates are similar. The inference one would draw is that a single factor or constellation of factors acting on both sexes increases in intensity as one moves eastwards round the Caspian. The excess incidence in women in the highest incidence areas noted in the earlier paper (Kmet and Mahboubi, 1972) is less marked with the present data from the extended period—the higher rate observed in Region 6b could reflect the diluting effect of a higher number of male migrants. However, the incidence is at least as high in women as in men, in striking contrast to many other areas where it is predominantly a male disease.

Some comment is needed on the methods of diagnosis. For many tumours microscopic confirmation of a diagnosis is necessary for a scientific investigation of incidence. The great majority of our oesophageal cancers were diagnosed radiologically, and for this tumour we feel that radiological diagnosis is likely to be correct in a high percentage of cases. Out of a sample of 128 patients who were traced 94 had died within 6 months of diagnosis, giving strong support to the initial diagnosis. For the 10 to 15% diagnosed solely on clinical grounds, a comparison can be made with the situation in the Transkei, where at the Oesophageal Cancer Clinic, of 1260 cases referred to the Clinic with dysphagia, only 115 (9%) were found not to have oesophageal cancer (Rose and Proctor, 1970). We feel, then, that no serious bias has arisen due to lack of histological confirmation. On the other hand, if we had relied solely on microscopically proven cases, the incidence pattern would have been totally false (see Table II).

For other tumours, the extent to which the figures given in the tables are affected by errors in diagnosis or underreporting is not known. However, certain points of interest emerge from the tables as they are which could warrant further investigation. In particular, stomach cancer is the second most common tumour in every region and varies little in incidence, except for a deficit in females in Gorgan. This uniformity in the incidence of stomach cancer is in very striking contrast to the incidence of oesophageal cancer, and is another feature of the registry experience which adds credence to the pattern of oesophageal cancer incidence.

Breast cancer varies considerably, being nearly 3 times higher in Gilan than in much of Mazandaran. This difference is the same whether one compares age adjusted, truncated or crude rates. More data are needed, however, before one can be sure of the magnitude of the differences.

There is an unusual number of tumours of the small intestine (Table III). Lack of histological diagnosis prevents us knowing if these are lymphomata, which would thus make the Caspian Littoral an extension of the middle east intestinal lymphoma belt. For tumours at other sites, either the numbers are too small or the doubts on the diagnosis are too large for comment to be useful at this stage. However, we would consider it unlikely that oesophageal cancer is the only aspect of the pathology of the region which reflects the great ecological variety.

### CONCLUSION

Given the suspected variation in oesophageal cancer incidence, it has proved feasible—in an area with but one physician for each 8000 inhabitants, no local pathology service, no large medical centre and with difficult communication—to organize a population based cancer registry adequate to reveal the extent and nature of the variation. It is felt strongly that such an approach could be revealing in other areas of the world, and for other tumours where similar gradients are suspected.

The regional pattern of variation described for oesophageal cancer parallels changes in certain ecological variables. Cancer incidence increases with a decline in rainfall and with the associated changes in soil types, natural vegetation and farming practice (Kmet and Mahboubi, 1972). We consider that the proper way to commence exploitation of the unique observational situation which exists in the study area is a population based investigation designed to determine the distribution, within and between regions of those features of man and his immediate environment which both explain the regional variation in risk and indicate where more detailed investigation of specific items might be rewarding.*

We would like to thank all the doctors in Mazandaran, Gilan and Ardebil who provided the basic information about the cancer patients; Dr Falati; Mr Deirmina and Mr Toulamy for their work in running the Caspian Cancer Registry; Dr Moussadeq and Mr Yarandpour of the Malaria Eradication Organization who gave us information on the ethnic composition of the villages in Gorgan and Gonbad; and Dr Muir of the IARC and Professor Sir Richard Doll of Oxford University for criticism of the text.

### REFERENCES

AHMED, N. & COOK, P. (1969) The Incidence of Cancer of the Oesophagus in West Kenya. *Br. J. Cancer*, **23**, 303.

BURRELL, R. J. W. (1962) Esophageal Cancer among Bantu in the Transkei. *J. natn. Cancer Inst.*, **28**, 495.

COOK, P. J. & BURKITT, D. P. (1971) Cancer in Africa. *Br. med. Bull.*, **27**, 14.

DOLL, R. & COOK, P. J. (1967) Summarizing Indices for Comparison of Cancer Incidence Data. *Int. J. Cancer*, **2**, 269.

HABIBI, A. (1965) Cancer in Iran: A Survey of the Most Common Cases. *J. natn. Cancer Inst.*, **34**, 553.

HAGHIGHI, P., NABIJADEH, I., ASWADI, S. & MOLALLATEE, E. A. (1971) Cancer in Southern Iran. *Cancer, N.Y.*, **27**, 965.

KMET, J. & MAHBOUBI, E. (1972) Oesophageal Cancer in the Caspian Littoral of Iran: Initial Studies. *Science, N.Y.*, **175**, 856.

ROSE, E. F. (1967) A Study of Esophageal Cancer in the Transkei. *Natn. Cancer Inst. Monog.*, **25**, 83.

TUYNS, A. J. (1970) Cancer of the Oesophagus: Further Evidence of the Relation to Drinking Habits in France. *Int. J. Cancer*, **5**, 152.

UICC (1966) *Cancer Incidence in Five Continents*, **1**. Ed. R. Doll, P. Payne and J. A. H. Waterhouse. Berlin, Heidelberg, New York: Springer-Verlag.

UICC (1970) *Cancer Incidence in Five Continents*, **2**. Ed. R. Doll, C. S. Muir and J. A. H. Waterhouse. Berlin, Heidelberg, New York: Springer-Verlag.

* Such a study is now under way, organized and supported jointly by the School of Public Health and the Institute of Public Health Research, University of Teheran, and the Food Nutrition Institute, Ministry of Health, Teheran, and the International Agency for Research on Cancer, Lyon, and supported by the Medical Research Council, London and the National Cancer Institute, Bethesda, USA.